# Assessing the antibiotic potential of essential oils against *Haemophilus ducreyi*

**DOI:** 10.1186/1472-6882-14-172

**Published:** 2014-05-27

**Authors:** Zachary Lindeman, Molly Waggoner, Audra Batdorff, Tricia L Humphreys

**Affiliations:** 1 Allegheny College Department of Biology, 520 N. Main St., Meadville, PA 16335, USA

**Keywords:** *Haemophilus ducreyi*, Chancroid, Essential oil

## Abstract

**Background:**

*Haemophilus ducreyi* is the bacterium responsible for the genital ulcer disease chancroid, a cofactor for the transmission of HIV, and it is resistant to many antibiotics. With the goal of exploring possible alternative treatments, we tested essential oils (EOs) for their efficacy as antimicrobial agents against *H. ducreyi*.

**Methods:**

We determine the minimum inhibitory concentration (MIC) of *Cinnamomum verum* (cinnamon), *Eugenia caryophyllus* (clove) and *Thymus satureioides* (thyme) oil against 9 strains of *H. ducreyi* using the agar dilution method. We also determined the minimum lethal concentration for each oil by subculturing from the MIC plates onto fresh agar without essential oil. For both tests, we used a 2-way ANOVA to evaluate whether antibiotic-resistant strains had a different sensitivity to the oils relative to non-resistant strains.

**Results:**

All 3 oils demonstrated excellent activity against *H. ducreyi*, with MICs of 0.05 to 0.52 mg/mL and MLCs of 0.1-0.5 mg/mL. Antibiotic-resistant strains of *H. ducreyi* were equally susceptible to these 3 essential oils relative to non-resistant strains (p = 0.409).

**Conclusion:**

*E. caryophyllus*, *C. verum* and *T. satureioides* oils are promising alternatives to antibiotic treatment for chancroid.

## Background

*Haemophilus ducreyi*, a Gram-negative coccobacillus, is a strict human pathogen responsible for the development of chancroid, which is a sexually transmitted infection (STI) that causes the formation of genital ulcers 4–10 days after acquisition of the bacteria and persists in some cases for 1–3 months. While relatively rare in the United States, chancroid is more prevalent in regions of Africa, Asia and Latin America due in part to the limited availability of health-care resources such as antibiotics. As there is no current vaccine for chancroid, treatment focuses on the use of antibiotics, typically 500 mg of ciprofloxacin administered orally twice a day for three days, although azithromycin, ceftriaxone, and erythromycin are also common [1].

Although no one strain of *H. ducreyi* demonstrates the wide range of and high degree of antibiotic resistance that is found in some bacteria, an increasing number of strains of *H. ducreyi* have developed some degree of antibiotic resistance [2]. Beginning in the 1970’s, strains of *H. ducreyi* emerged that possessed resistance to penicillin and its derivatives [3]. Subsequently, strains of *H. ducreyi* demonstrated resistance to sulfonamides, aminoglycosides, tetracyclines, and chloramphenicol [4]. The development of antibiotic resistance in *H. ducreyi* is of particular concern given the connection between HIV-1 and chancroid [5]. Genital ulcer diseases (GUDs), which include chancroid, are a known cofactor for HIV transmission; people with a GUD are 50–300 times more likely to contract HIV per unprotected act of vaginal intercourse. In many areas where HIV is prevalent, chancroid is the most common GUD [5]. Therefore, treatment of chancroid seems likely to help prevent the spread of HIV, especially in impoverished regions of the developing world with poor health care institutions.

With the increasing resistance of strains of *H. ducreyi* to antibiotics currently in use and the threat that chancroid contributes to the spread of HIV, it seems prudent to search for alternatives to the current methods of treatment for chancroid. One such alternative that has shown promise in the treatment of other bacteria but has never been tested on *H. ducreyi* is the use of essential oils, a chemically diverse group of plant-derived compounds, many of which have antibacterial properties [6]. Importantly, the antibacterial activity of essential oils is as potent against bacterial strains with antibiotic resistance as their non-resistant counterparts [7]. For example, MRSA is susceptible to thyme white oil, lemon oil, lemongrass oil, and cinnamon oil [7]. The susceptibility of STIs to essential oils is not well studied, although *Neisseria gonorrhoeae* is susceptible to the essential oil of *Croton gratissimus*[8].

Based on the antibacterial effects of a wide variety of essential oils, we determined the minimum inhibitory concentrations (MICs) and minimum lethal concentrations (MLCs) of *E. caryophyllus*, *C. verum* and *T. satureioides* using the agar dilution method [7,9]. Based on our results, *H. ducreyi* is more susceptible to *C. verum*, *T. satureioides* and *E. caryophyllus* than any of the control organisms, making all of three oils excellent candidates for clinical trials in future studies.

## Methods

### Selection of bacterial species

Nine strains of *Haemophilus ducreyi* were chosen to reflect the varying degrees of antibiotic resistance found clinically: 35000HP, CIP542, HMC46 (Pc^R^, Tc^R^, Cm^R^), HMC48 (Pc^R^, Tc^R^, Cm^R^), HMC56 (Pc^R^, Tc^R^), HMC88 (Pc^R^, Tc^R^, Cm^R^, TMP^R^, Str^R^, Kan^R^), HMC49, HMC53 (Pc^R^, Tc^R^, Cm^R^) and HMC112 [10]. In addition, *Escherichia coli* K-12, *Pseudomonas aeruginosa* 49189 (ATCC, Manassa, VA, USA)*, Lactobacillus reuteri* HM-102 (BEI Resources, Manassas, VA, USA)*,* and *Staphylococcus aureus* strain +4651 (Presque Isle Cultures, Erie, PA, USA) were used as controls. All strains of *H. ducreyi* were provided courtesy of Stanley Spinola (Indiana University School of Medicine) or Patricia Totten (University of Washington). *S. aureus* and *E. coli* were chosen as positive controls because all selected essential oils are effective bacteriostatic agents against both [9]. *P. aeruginosa* was chosen as a control representing relative resistance to essential oils, being resistant to most essential oils except cinnamon and oregano [9]. *L. reuteri* was chosen as a representative of the many *Lactobacillus* species that make up the vaginal flora. The susceptibility of *L. reuteri* is unknown, although *Lactobacillus* strains are resistant to tea tree oil concentrations up to 2.0% [11].

### Essential oils tested

The following pure essential oils were selected for analysis: *Eugenia caryophyllus* (clove)*, Cinnamomum verum* (cinnamon)*,* and *Thymus satureioides* (thyme). All essential oils were purchased from NOW Foods (Bloomingdale, IL, USA).

### Determination of minimum inhibitory concentrations (MICs)

MICs were determined by the agar-dilution method based on the Clinical and Laboratory Standards Institute guidelines [6,8]. Each essential oil was diluted in sterile 0.15% agar solution [9]. Twofold serial dilutions of each essential oil were performed to create dilutions of each essential oil ranging from 2.5% to 0.001% (mg/mL varies by density of each oil). Chocolate agar plates were inoculated and incubated as previously described.

*L. reuteri* was grown at 33°C in a 10% CO_2_ incubator. All other control organisms were grown at 37°C. After the initial screen, the range of concentrations tested in subsequent assays was narrowed down to the three concentrations nearest to the apparent MIC. Each strain/concentration combination was tested in triplicate for each assay, and the entire assay was repeated three times for each of the strains of *H. ducreyi* tested, as well as for all control organisms. MICs were defined as the lowest concentration of oil for which there was no visible bacterial growth on any of the plates assayed [9].

### Determination of minimum lethal concentrations (MLCs)

Subcultures were taken from each of the chocolate agar plates used in the MIC assays that exhibited no growth after 36 hours. A sterile wire loop was used to scrape the surface of the agar and any bacteria present were thus transferred to a fresh chocolate agar plate that lacked any essential oil and incubated for 24–72 hours. The lowest concentration of an essential oil from which the bacteria failed to grow within 24–72 hours (depending on the relative normal growth rates of each strain of bacteria tested) after being transferred to a new chocolate agar plate was defined as the MLC.

### Data analysis

Each phase of this study was performed in triplicate. Mean values for the MICs and MLCs were calculated for each essential oil. A 2-way ANOVA was used to analyze the differences in MIC and MLC between resistant and non-resistant strains. Differences in values of p < 0.05 were considered significant. Data were analyzed with IBM SPSS Statistics, Version 19 (IBM Corporation, Armonk, NY).

## Results and discussion

MICs were determined for *C. verum*, *E. caryophyllus*, and *T. satureioides* using the agar dilution method. *E. caryophyllus* had the most potent antibacterial effects out of the essential oils tested, with an MIC of 0.085 ± 0.03 mg/mL (mean ± SD) and an MLC of 0.14 ± 0.07 mg/mL (mean ± SD). However, both *C. verum* and *T. satureioides* were relatively effective as well, with MICs of 0.23 ± 0.14 mg/mL and 0.29 ± 0.11 mg/mL (mean ± SD), respectively (Table 1). According to Ríos and Recio’s criteria [6], *E. caryophyllus* is the most promising oil as the MIC and MLC for most strains is at or below 0.1 mg/mL. Interestingly, all of the control organisms had at least 10-fold higher MICs and MLCs than any of the strains of *H. ducreyi* for each of the oils tested, suggesting that these essential oils may be particularly effective against *H. ducreyi* (Table 1). In the MLC assay, the antibiotic resistant strains of *H. ducreyi* were equally susceptible to the essential oils as the strains without antibiotic resistance (p = 0.409), as is the case with previous studies [7,9], (Table 1). There was no interaction between the type of oil and antibiotic resistance (p = 0.227).

**Table 1:** **MICs**^**a **^**and MLCs**^**b **^**of essential oils against antibiotic resistant and sensitive strains of *H. ducreyi***

	***E. caryophyllus***		***C. verum***		***T. satureioides***	
**Organism**	**MIC**	**MLC**	**MIC**	**MLC**	**MIC**	**MLC**
***H. ducreyi***						
35000HP	0.104	0.260	0.103	0.260	0.460	0.460
HMC112	0.050	0.104	0.103	0.260	0.230	0.230
HMC46^c^	0.050	0.104	0.520	0.520	0.230	0.460
HMC48^c^	0.104	0.104	0.260	0.520	0.230	0.230
HMC56^c^	0.050	0.104	0.260	0.260	0.230	0.460
HMC88^c^	0.104	0.104	0.260	0.260	0.230	0.460
HMC49^c^	0.104	0.104	0.260	0.260	0.460	0.460
HMC53^c^	0.104	0.260	0.260	0.260	0.230	0.230
CIP542ATCC	ND^d^	ND^d^	0.050	0.050	ND^d^	ND^d^
**Controls**						
*E. coli*	2.6	2.6	25.8	25.8	4.6	4.6
*S. aureus*	2.6	2.6	10.3	25.8	4.6	4.6
*P. aeruginosa*	5.2	10.4	5.2	5.2	9.2	9.2
*L. reuteri*	≥2.6	≥2.6	5.2	10.3	4.6	4.6

MIC and MLC are reported in mg/mL. For all strains shown, n = 3.

^a^Minimum inhibitory concentration (MIC) is the lowest concentration of oil-infused agar on which no growth occurred.

^b^Minimum lethal concentration (MLC) is the concentration of oil-infused agar from which no growth occurred after transfer to fresh agar without essential oil.

^c^Denotes resistance to penicillin, tetracycline, kanamycin, and chloramphenicol.

^d^Not done; due to the extremely fastidious growth requirements of this strain, sufficient growth was not obtained to determine the MIC and MLC for these oils.

Based on the results of this study, the essential oils of *C. verum*, *T. satureioides*, and *E. caryophyllus* all are potent bactericides against *H. ducreyi*. Therefore, any of these three essential oils would be a promising alternative to antibiotics. However, although the effectiveness of all essential oils tested varied based on the strain of *H. ducreyi*, for each strain we tested *E. caryophyllus* oil had either the lowest MLC or was tied for the lowest MLC with either *C. verum* or *T. satureioides* (Table 1). Therefore, *E. caryophyllus* oil in particular provides an excellent opportunity for further study.

The viability of *E. caryophyllus* oil as a potential treatment for chancroid is further supported by the results we obtained for our control organisms, particularly *L. reuteri. Lactobacillus* species are a large component of the flora in the female reproductive tract and play an important role in protecting the vaginal environment from invasion by foreign pathogens [12]. This is especially important in the treatment of chancroid because disrupting the *Lactobacillus* species in the vagina can increase the susceptibility to HIV [13]. Therefore, a suitable potential treatment for chancroid would ideally be harmless to natural flora. Although *L. reuteri* is a resident of the gut, rather than vagina, it was chosen as a representative of the *Lactobacillus* species that inhabit the vagina because it grows most readily on chocolate agar. Importantly, we found that *L. reuteri* is much more tolerant (at least tenfold higher MLCs for each oil) of *C. verum*, *T. satureioides*, and *E. caryophyllus* oils than *H. ducreyi* (Table 1). It is therefore likely that MLC doses of these three oils for *H. ducreyi* would have no adverse effects on host flora.

Another quality of essential oils that would make them a good alternative treatment for chancroid is their low cytotoxicity. Because chancroid is a genital ulcer disease, topical administration appears to be the most likely choice. When administered topically, essential oils have low toxicity provided they are diluted to at least 3%-4% in carrier oil [14]. Possible side effects of essential oils applied to the skin are confined to irritation or allergic reactions [15]. However, because the MLCs for *C. verum*, *E. caryophyllus*, and *T. satureioides* range from 0.01-0.05% (ca. 0.1-0.5 mg/mL), it is unlikely that such side effects would occur (Table 1).

## Conclusions

Based on the results of this study, *C. verum* (cinnamon), *T. satureioides* (thyme), and particularly *E. caryophyllus* (clove) oil would all make excellent candidates for an alternative treatment for chancroid that would be relatively risk free and cheaply produced. We recommend further study to assess the antibacterial activity of these oils in vivo.

## Abbreviations

Cm^R^: Chloramphenicol-resistant; GUD: Genital ulcer disease; Kan^R^: Kanamycin-resistant; HIV: Human immunodeficiency virus; MIC: Minimum inhibitory concentration; MLC: Minimum lethal concentration; MRSA: Methicillin-resistant *Staphylococcus aureus*; Pc^R^: Penicillin-resistant; STI: Sexually-transmitted infection; Tc^R^: Tetracycline-resistant; TMP^R^: Trimethoprim-sulfamethoxazole-resistant.

## Competing interests

The authors declare that they have no competing interests.

## Authors’ contributions

ZL participated in the study design, data collection, statistical analysis, preparation and revision of the manuscript. MW participated in the study design, data collection and analysis. AB participated in collection and analysis of the data. TH participated in the study design, interpretation of the data and manuscript revision. All authors read and approved the final manuscript.

## Pre-publication history

The pre-publication history for this paper can be accessed here:

http://www.biomedcentral.com/1472-6882/14/172/prepub
